# The Role of Religion, Spirituality and Social Media in the Journey of Eating Disorders: A Qualitative Exploration of Participants in the “TastelifeUK” Eating Disorder Recovery Programme

**DOI:** 10.1007/s10943-023-01861-0

**Published:** 2023-07-09

**Authors:** Barbara Mitra, Diana Archer, Joanne Hurst, Deborah Lycett

**Affiliations:** 1 Joint Head of English, Media and Culture, University of Worcester, City Campus, Worcester, WR1 3AS UK; 2 Tastelife, Kenilworth, UK; 3 School of Nursing, Midwifery and Health, Coventry University, Priory Street, Coventry, CV1 5FB UK; 4 Institute Director for Health & Wellbeing, Centre for Healthcare and Communities, Coventry University, Coventry, CV1 5FB UK

**Keywords:** Eating disorders, Spiritual struggles, Faith, Religion, Qualitative study, Social media, Media, Body image, Anorexia nervosa, Bulimia nervosa

## Abstract

This study explores the religious and spiritual aspects of eating disorder recovery and the role of social media in the context of a third sector community-based recovery group in the UK. Four online focus groups explored participant perspectives (17 participants in total) using thematic analysis. The qualitative findings highlight that relational support from God is important in eating disorder recovery and coping, although this can be challenged by spiritual struggles and tensions. Relational support from people is also relevant where it offers a place to share different experiences together giving a sense of community belonging. Social media was also found to be important in relation to eating disorders, either providing a community of support or exacerbating existing issues. This study suggests that the role of religion and social media should be acknowledged where it is important for that individual in relation to eating disorder recovery.

## Introduction

As an umbrella term, eating disorders can include a wide range of food behaviours ranging from anorexia nervosa, bulimia, binge-eating disorders, other eating or feeding disorders not otherwise specified (see National Eating Disorders Association, 2022) and avoidant/restrictive food intake disorders (National Health Service, 2021). According to the Priory Group (2022) between 1.86% and 9.5% of the UK population have an eating disorder and are most common in individuals between 16 and 40 years old but often develop during adolescence. Recent research from the National Health Service suggests that up to 6.4% of adults display signs of an eating disorder (BEAT, 2022).

The aetiology of eating disorders is multi-factorial with known bio-psycho-social determinants and whilst many different factors can affect the development of an eating disorder (Blodgett Salafia et al., 2015) the conditions may share similarities (Hauck et al., 2020). Genetic components with inherited personality traits (perfectionism, sensitivity and obsessional) can play a key role, alongside temperament, formative relationships and early life experiences (Thornton et al., 2011). Negative affect, body dissatisfaction and dieting are significant contributing factors (Rosenvinge & Pettersen, 2015; Stice et al., 2018), often perpetuated by family, peer group and media (including social media) and pressures to be thin (Perloff, 2014).

Religion and spirituality have received increasing attention as factors that shape and influence attitudes and behaviours in relation to the body (see Strenger et al., 2016) and are recognised “as important aspects of eating disorders” (Smith et al., 2004: 172). Akrawi et al., (2015: 2) define religiosity as “a system of organised beliefs, practices, rituals and symbols” that enable closeness to the transcendent, whereas spirituality is “the personal quest for understanding answers to ultimate questions about life.” Religion is organised around a set of beliefs and practices whereas spirituality, whilst associated with religion, is an interconnection of something beyond ourselves and connecting something within ourselves (Victor & Treschuk, 2020). Religious views of the body might influence perceptions of one’s body as sacred (Homan & Cavanaugh, 2013) or inspire feelings of shame (Goulet et al., 2017). Focus on divine struggles including “negative thoughts or feelings” about God (Exline et al., 2016: 8) can include anger, disappointment and guilt around binge eating (Smith et al., 2004) but equally can include ideas of being saintly in relation to fasting and purging (Forthun et al., 2003:9).

Religion, particularly a relationship with the divine, can provide some protection against developing an eating disorder acting as a buffer to coping with difficulties (Geller et al., 2018) but at the same time can actually exacerbate existing issues. This depends on individual perceptions relating to religious teachings about the nature and purpose of the body (as a sacred gift or needing control of carnality), as well as teachings about the nature of the self (as a valued child of God versus a sinner) (Oman, 2018; Spangler, 2010). This may relate to religiousness characterised by either intrinsic or extrinsic religious motivations. Intrinsic religious motivation is the internalisation of religious beliefs into everyday life whilst extrinsic religious motivation is where religiosity serves other ends such as status (Akrawi et al., 2015). Forthun et al. (2003) suggest that intrinsically oriented individuals are likely to use a more collaborative style of coping, communing with the divine and having a more secure attachment to God. This secure attachment helps to develop “an internal sense of worth and not being dependent on others for approval” (Homan & Cavanaugh, 2013: 1531) which relates to body image concerns.

Much of the research on religion and eating disorders has been predominantly quantitative (Exline et al., 2016; Forthun et al., 2003; Geller et al., 2018; Goulet et al., 2017; Smith et al., 2004; Strenger et al., 2016) and much research has been conducted in the US. These studies often draw on undergraduates particularly from private American Christian colleges (See Homan & Boyatzis, 2010; Homan & Cavanaugh, 2013; Cottingham et al., 2014) or with patients from eating disorder clinics (see Richards et al., 2007) as in-patients (Malson et al., 2011). Findings from these studies reveal both the “positive and negative associations with disordered eating and psychopathology” (Akrawi et al., 2015:4) focusing on attachment to God (Strenger et al., 2016) and divine struggles in relation to eating disorders (Exline et al., 2016). Those studies that focus on specific eating disorders often tend to explore anorexia or bulimia (see Stammers, 2020) or focus on a specific population (such as undergraduates see Smith et al., 2004). Studies that draw on undergraduates or college students often use surveys or self-reporting data (Jarman et al., 2021) such as the Eating Attitudes Test, Religious Orientation Scales (Smith et al., 2004) and The Eating Disorder Diagnostic Scale (Strenger et al., 2016). Instead, our research uses a qualitative research method to illuminate experiences (Hammarberg et al., 2016; Marsden et al., 2007) and to enable deeper understandings of what the religious and social media struggles look like to those living with eating disorders and to explore whether religious support is desired in recovery programmes. Our research therefore links religiosity and eating disorders with the wider community, including religious communities but also places these issues into the context of social media. This is important as social media itself has grown at a rapid pace in the twenty-first century and is now part of wider cultural contexts (Lee, 2018).

Social media platforms make it easier for messages to spread and for pressures about body image to permeate (Fardouly & Vartanian, 2016; Mabe et al., 2014; Wick & Keel, 2020). With content available anytime and anywhere the dominance of visual content (Knoblock-Westerwick et al., 2014) can encourage upward comparisons (Burnette et al., 2017) where others are deemed better than oneself. Shame and comparison on social media reinforce the body as a key site of “identity, empowerment and control” (Beattie et al., 2019:18) with self-surveillance continually being reinforced, along with increases in appearance concerns (Spettigue & Henderson, 2004; Wick & Keel, 2020). There are eating disorders sites with stories about purging and BMIs and advice on how to lose weight (Brotsky & Giles, 2007:106) and posts that can induce feelings of body-related shame (Carrotte et al., 2015). Tiggemann and Anderberg (2019) asked participants to view different images on Instagram and self-report their responses using various scales to assess body dissatisfaction. Tiggemann and Anderberg (2019) themselves noted, that their participants (from the U.S.) may have viewed images differently to how they would have done at home (and the images viewed on Instagram were only viewed for less than five minutes). There is a lack of longitudinal data and causality cannot be assumed regarding social media use.

Wilksch et al. (2020) suggested that social media platforms with a strong focus on posting and viewing images can be associated with elevated disordered eating (although their research only included adolescents). They detailed how social media might encourage behaviours such as following strict meal plans, strict exercise and over-evaluations of shape and weight. Padin et al. (2021) highlight that time spent on image-related social media is associated with problematic eating behaviours including lower self-esteem. Exposure to social media, where comparisons are encouraged, can exacerbate internalised idealised body images and promote body image dissatisfaction (Mitra, 2017). Studies on Facebook have found a correlation between Facebook use and eating disorder psychopathology, including increased weight and shape concerns (Basterfield et al., 2018), although relationships between self-esteem and social media is actually complex (Saiphoo et al., 2020: 5). Some social networking sites revolve around images including “physical appearance and self-representation” (Mingola et al., 2017: 2) where people upload their own images. Content on social media ranges from “perfect bodies” (Tiggemann & Anderberg, 2019:3) to food, lifestyle, health and fitness posts (Carrotte et al., 2015). Many additional studies have explored the relationship between social media, body image and eating disorders (Basterfield et al., 2018; Karsay et al., 2021; McClean et al., 2015; Saffran et al., 2016; Tiggemann & Anderberg, 2019; Williams & Ricciardelli, 2014) although few studies also included religiosity/spirituality alongside.

However, as with religiosity, social media can also provide support groups which buffer against eating disorders (Basterfield 2018; Weinstein, 2018) fostering a sense of belonging and support. Williams and Ricciardelli (2014) found that supportive posts, support groups and messages of encouragement can help buffer against body image concerns and can also highlight the artificiality of media images (Burnette et al., 2017). Additionally, such comparisons may be automatic and happen at an unconscious level (Chatard et al., 2017) which can make social media difficult to counteract. Some studies focused exclusively on women (Meier & Gray, 2017; Slater & Tiggemann, 2015) some on adolescents (Slater & Tiggemann, 2015) and many studies were conducted in North America (Padin et al., 2021). Members can be anonymous whilst posting and receiving support in some online groups. Kendal et al., (2017: 105) looked at an eating disorder charity website which also included a discussion forum and found that “for people in recovery, the forum appeared to help by facilitating mutual support and encouragement.” They also noted that peer support may be attractive because of mutual understandings and help for those seeking recovery (Brotsky & Giles, 2007). Teufel et al. (2013) noted that support is often social support rather than professional support online which can make the situation complex. Whilst negative emotions can arise due to comparisons and peer judgements, there can also be social connectedness and validation online (Weinstein, 2018). Our research explores the links between social media, body image and religiosity in individual recovery journey contexts.

### Aim

To explore the religious and spiritual aspects of eating disorders discussed within, and important to, those attending an eating disorder recovery programme (tastelifeuk.org) including the use of social media.

### Objectives

To conduct a qualitative semi-structured focus group study to:

1. Explore religious and spiritual issues (including both religious struggles and religious coping) important to those attending tastelife UK programmes.
2. Explore participant experience of discussing these issues on the tastelife UK programme.
3. Gain participant perspectives of how religious issues should be addressed on the tastelife UK programme.
4. Explore the role of social media in tastelife UK participants and the relationship with religious and spiritual issues.

## Methods

### Design

In line with other research such as Basterfield et al. (2018) who used focus groups with 13 individuals, we conducted focus groups with 17 individuals living with eating disorders (PLD), carers (C) and volunteers (V) who had participated in a tastelife programme (see Marchetti-Mercer, 2012; Reczek, 2014). The focus group involved in-depth discussions guided by our research aims (Palmberg et al., 2014) to tease out complex interactions and contexts relating to eating disorders in a flexible manner (Bryman, 2016) and we expand the research in this area by linking social media, religiosity and eating disorders. See Table 1 for focus group questions.

**Table 1:** Focus group questions

Focus	Questions	Sub questions (prompts) if needed
Relating to the tastelife course	Why did you choose to take part in tastelife?	Have you sought help before/How did you find out about the course?
	What has been most useful about taking part in this course	Was there a particular session that was particularly useful?
	What has been least useful about taking part in this course?	Was there a session/topic that would have been good to have included?
Questions relating to spirituality/faith	What do you understand by the terms spirituality	Would you like to share awareness of personal spirituality or faith in light of these discussions?
	What do you understand by the term faith	How does spirituality/faith impact on your self-image/self-worth or how you view yourself?
	What do spirituality/faith mean to you personally	Can you give some examples?
	Has faith/spirituality played a role in relation to your eating disorder	Has your spirituality/faith helped you in relation to your eating disorder? Examples?
	Has faith/spirituality played a role in relation to carers/those supporting people with eating disorders	Has your spirituality/faith hindered you in relation to your eating disorder? Examples?
		How has your faith/spirituality helped those you are supporting those with eating disorders?
		How has faith/spirituality hindered those you are supporting with eating disorders?
		Have people in your faith/spiritual community been supportive or not? Examples?
Questions in relation to social media	What social media do you participate in personally	Have people in your social media networks been supportive? How?
	Has social media played a role in relation to your eating disorder?	Have people in your social media networks been unsupportive? How?
	Has social media played a role in relation to carers/those supporting people living with eating disorders	What role has social media played/Examples?
	Does social media play a role in relation to your faith/spirituality (if faith/spirituality is mentioned)	Has social media helped you in relation to your eating disorder? Examples?
		Has social media hindered you in relation to your eating disorder? Examples?
		How has social media helped regarding those you are supporting with eating disorders?
		How has social media hindered regarding those you are supporting with eating disorders?
		How does faith/spirituality relate to the eating disorder for those living with eating disorders/those caring for people with eating disorders?

### Ethical Approval

Ethical approval was given from Coventry University Research Ethics Committee (P88667) and was ratified by the University of Worcester. The paper is reported in accordance with the Consolidated Criteria for Reporting Qualitative Research. The focus groups were recorded using Microsoft Teams and once transcribed, the recordings were deleted. The transcribed materials contained no identifiable details. Participants were informed about the research and informed consent was obtained and they were informed that data would be anonymised and kept confidential, and that the material would be used in relation to publications.

### Setting

Tastelife UK is a charity set up to offer hope for people living with eating disorders and to help their supporters through an eight-session community self-help course. The course is evidence based and reflects Christian values of health and wholeness. It is fully adaptive for, and sensitive to, those of all faiths and none. It welcomes all ages, including carers and concerned friends. Anyone aged under 18 years must be accompanied by an adult who has participated in a tastelife programme in some capacity (person living with an eating disorder, carer, volunteer). The focus of our research includes the interplay of religion, social media and communities in the lives of participants on tastelife programmes.

### Participants/Eligibility Criteria

Three groups of participants were recruited purposively according to the following eligibility criteria (see Table 2). Particular attention was given to the vulnerability of participants and researchers ensured that continued consent and assent was present throughout the focus group. Participants were sign-posted to further support to discuss issues raised in the focus groups as required. The focus groups were confidential but participants were made aware that researchers were obliged to disclose information which indicated that the participant or others would be at risk of maltreatment (National Institute for Health & Care Excellence, 2017a). Vigilance was applied as described by National Institute for Health & Care Excellence (2017b) for the management of eating disorders. See Table 2 Eligibility Criteria.

**Table 2:** Eligibility criteria

Inclusion criteria	Exclusion criteria
1. Either be an individual reporting some aspect of disorder eating/eating disorder (which may or many not have been medically diagnosed) or involved in the care of someone struggling in this way	1. Those who appear distressed at the prospect of having a conversation with a researcher
2. Have attended a tastelife UK programme in the past	2. Those who are currently sectioned under the Mental Health Act 1983 will not be considered as they lack capacity to provide consent
3. Able and willing to provide consent and those under 16 must be Gillick Competent	3. Those currently receiving treatment for eating disorders as inpatients will be considered too unwell to participate

### Recruitment

Participants were recruited through tastelife UK who acted as a gatekeeper for participants on the study. Tastelife identified suitable individuals who had taken part in the course and invited them to take part in a focus group. Tastelife arranged a convenient time and date for interested individuals to meet with a researcher who provided participant information and invited individuals to complete a consent form. Those between 16 and 18 years were invited to complete an assent form.

### Research Team

The research team consisted of four researchers including the director of tastelife alongside three academics from differing disciplines. These comprised of a Professor in Religious Health Interventions with a clinical background as a dietitian: an Assistant Professor (co-facilitator) in the School of Nursing, Midwifery and Health with an Occupational Therapy focus and personal experience of an eating disorder; and a Principal Lecturer (focus group facilitator) in the Department of English, Media and Culture with a focus on social media and experience of qualitative research (but no personal experience of eating disorders). The director of tastelife founded the charity and community-based course as an outcome of having daughters with eating disorders. All the research team are Christians and have an ethos that values others (of all faiths and none), reflecting tastelife’s ethos of hope.

### Data Collection

The focus group facilitator (BM) and co-facilitator (JP) conducted all the focus groups. BM has a wealth of experience of conducting qualitative research methods (Mitra et al., 2014, 2018) and had no personal experience of eating disorders and had not been involved in tastelife and was interested in generating discussions about the research questions. BM has not researched religiosity or eating disorders previously. JP was present as the tastelife contact, having been a volunteer and also having had an eating disorder themselves in case anyone needed to debrief after the focus groups. The focus groups were conducted on Microsoft Teams (during COVID-19) which BM had become very adept at using during lockdowns. BM transcribed all four focus groups.

The focus groups lasted approximately between 75 to 90 min as the participants were keen to contribute and discuss their experiences. During the focus groups BM verbally summarised the key points covered to establish whether they had correctly interpreted the participant responses. This allowed “immediate” member checking where participants could revise or clarify their views in line with COREQ (Tong et al., 2007).

### Data Analysis

**Fig. 1:**
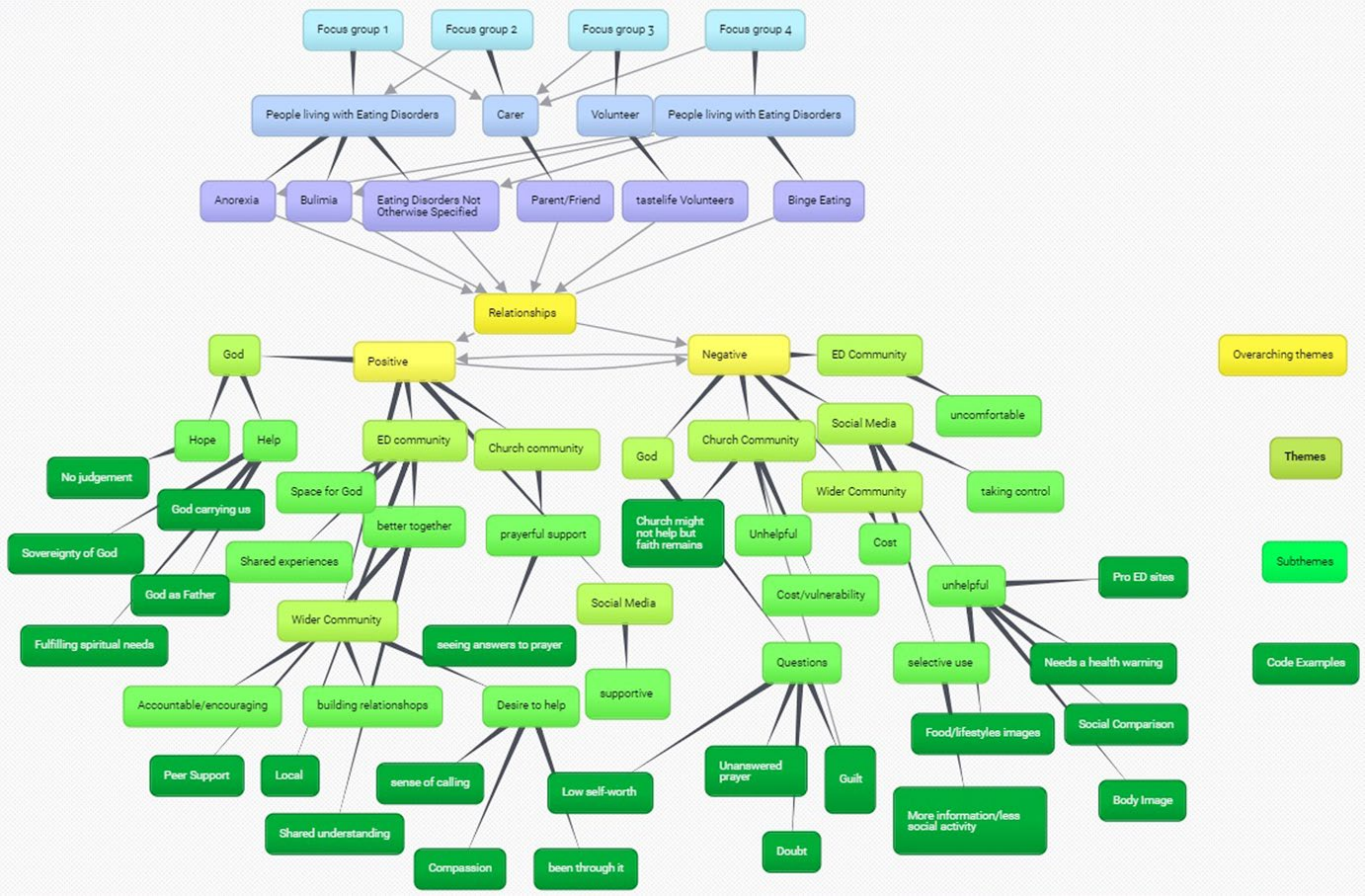
Coding and themes mind map

The team followed the guidance set out by Braun and Clarke (2006: 96) beginning with transcription and the four researchers then began the coding process (see the coding and Themes mind map (Fig. 1). Once the codes had been thoroughly reviewed, each team member read and re-read a focus group before cross-checking. BM read and re-read all four focus groups in relation to the themes that emerged. The team continued to meet at least once a month to check both coding and the themes/sub-themes. The whole team were thoroughly involved in this process through continual discussion of themes/sub themes to ensure consistency. If there were disagreements, each team member went back to the transcribed focus groups for consideration and met again to discuss and resolve such issues. Quotes were attributed to the themes and sub themes and the research team continued to meet to discuss implications and findings. BM as the lead researcher ensured that the team continued to meet and discuss interpretations to ensure that analysis was data driven. It was when no new topics were being raised in the research meetings that saturation (which is difficult to define in qualitative research—see Fusch & Ness, 2015) was surmised. Thematic analysis has been widely used to “identify, analyse and report themes within and across data by organising data and providing rich description” (Palmberg et al., 2014: 1407). It is a flexible approach (Griffiths et al., 2011: 169) which has been used to examine “underlying assumptions, conceptualisations and ideologies” (Maguire & Delahunt, 2017: 3352) in qualitative research. It allows for “identifying, analysing and reporting patterns (themes) within data” (Braun & Clarke, 2006: 6) enabling recurring responses that relate to our research aims to be discerned (Palmberg et al., 2014). The interview transcripts were analysed separately and coded inductively at the semantic level. After initial semantic coding the team then moved to discuss latent themes to move beyond descriptions and to identify hidden meanings, “underlying assumptions, ideas or ideologies that may shape or inform the descriptive or semantic content of the data” (Byrne, 2022: 1397). After initial transcription of the focus groups, contributions were also divided into those living with an eating disorder, carers and volunteers in order to assist with triangulation of data**.** The researchers adopted a realist approach wherein the participant’s experiences were taken at face value regarding their experiences of participating in the programme. Transcripts were read and re-read, and line-by-line coding was completed. The coding process moved back and forth across the dataset in an iterative process where comparisons were made between codes and phrases. Those with similar context or concepts were grouped together. Themes were illustrated by non-identifiable, verbatim quotes.

## Results

Originally, we aimed to have three focus groups consisting of 6–10 participants. Due to COVID-19 lockdowns, we conducted the focus groups online and conducted four focus groups. Focus groups 1 consisted of 5 participants (2 carers, 3 people living with eating disorders (one of whom was also a volunteer). Focus group 2 consisted of 6 people living with eating disorders. Focus group 3 consisted of 3 participants (1 carer and 2 volunteers who also had lived experiences of an eating disorder) and focus group 4 consisted of 3 participants (1 carer and 2 people living with an eating disorder). For the participants living with an eating disorder (PLD), 5 identified as having anorexia (AN), 3 binge eating (BED), 1 Bulimia (BN) and 1 described their eating disorder as overeating (OE). Out of those caring or supporting others (C), 3 were doing this in relation to anorexia and 1 was for an eating disorder not otherwise specified (OSFED). The two (out of three) volunteers also had an eating disorder (V&PLD), 1 identified as having bulimia and 1 as obesity (OB). The third volunteer did not have an eating disorder (NED) (see Table 3 for details of eating disorders and participants). Focus group 2 did not have carers or volunteers present.

**Table 3:** Participant demographic characteristics

Age ranges of participants	16–18 yrs. = 1;
	20–29 yrs. = 1;
	30–39 yrs. = 2;
	40–49 yrs. = 6;
	50–59 yrs. = 6;
	60 and above = 1
Gender	Male = 2 (FG1, C11, A) and FG4, C14, A)
	Female -15
Carer/volunteer/PLD	People living with Eating Disorders (PLD) = 10 (numbered 01–10)
	Carers (C) = 4 (Numbered 11–14) are those caring for someone with an ED
	Volunteer (V) = 3 (2 of these were also living with an eating disorder identified as V&PLD) (Number 15–16), V (Number 17)
Ethnicity	White British = 14;
	Scottish = 1;
	Northern Irish = 1;
	English = 1
Spiritual beliefs	Yes = 17
	1 Buddhist,
	16 Christian
Occupations	NHS and health related = 5; Social Worker = 1
	Charity fundraiser = 1; Gardener (P/T) = 2
	Tutor/lecturer = 2; Baker = 1
	Semi-retired = 1; Student = 1
	No employment = 4
	(Some respondents had more than 1 occupation)
Type of eating disorder (in words of the participant)	Anorexia = AN
	Bulimia = BN
	Overeating = OE
	Obesity OB
	Binge Eating = BED
	Other Eating Disorder Not Specified = OSFED
	No Eating Disorder noted = NED
Focus group number	FG = Focus group (FG1, FG2, FG3, FG4)
Focus Group 1	(FG1, PLD01, AN), (FG1, PLD, BN), (FG1, C11,AN Male), FG1 C12, AN), FG1, V& PLD,OB)
Focus Group 2	(FG2, PLD03, OB), (FG2, PLD04 AN), (FG2, PLD05, BED), (FG2, PLD06, AN), (FG2, PLD07, AN), (FG2, PLD08 BED),
Focus Group 3	(FG3, C13, OSFED), FG3 V16&PLD, BN) (FG3, V17, NED)
Focus Group 4	(FG4, PLD09, AN), (FG4, PLD10, BED), (FG4, C14, AN Male)

FG – Focus group; PLD = person living with an eating disorder; C = carer; V = volunteer

### Findings: Thematic Analysis

Codes were grouped into themes and subthemes, examples illustrating how these were formed is contained in Fig. 1.

Five main themes with associated subthemes were drawn from the data in answer to the research questions. All themes involved relationships in various forms (Table 4).

**Table 4:** Themes and subthemes

Theme	Theme 1: Experiencing relational support from God: Hope, help and questions (the anchor in the storm)	Theme 2: Experiencing relational support from people: Same storm but sometimes a different boat	Theme 3: Experiences of a church community: A mixed bag	Theme 4: The community and beyond: A ripple effect	Theme 5: Experiencing relational support from social media: "it should come with a health warning"
Subtheme	Subtheme 1: Hope: God is bigger and always there	Subtheme 1: Valuing shared experiences	Subtheme 1: Care and compassion: Prayerful support	Subtheme 1: Accountable and encouraging	Subtheme 1: Helpful/positive: Perception versus reality
Subtheme	Subtheme 2: Help: God can act	Subtheme 2: Better together	Subtheme 2: Vulnerability: The risk and cost of being real	Subtheme 2: Communication and inclusion: Building relationships	Subtheme 2: Unhelpful/negative aspects
Subtheme	Subtheme 3: Questions for God	Subtheme 3: Making space for the presence of God: Exploring spirituality and faith in recovery	Subtheme 3: Best intentions but often unhelpful approaches	Subtheme 3: A desire to help others	Subtheme 3: Selective use

## Theme 1: Experiencing Relationship Support from God

Participants discussed experiences which reflected being in relationship with “a creator God who cares for me personally” (FG4, C14, AN) and this relationship offered support in the recovery journey. One person noted: “It’s only with the help of God that I can do it” although they also struggled with feelings of “letting Him [God] down” (FG2, PLD04, AN). These ideas were articulated through themes of hope and help taking into account personal struggles and complexities of a relationship with God.

## Theme 1 Subtheme 1 Hope: God is Bigger and Always There

Hope was expressed through God being present and participants being able to draw on their faith in God, as one participant commented they could leave their eating disorder “in God’s hands” (FG2, PLD07, AN). God is perceived as something bigger than the eating disorder which contributed to hope for recovery. “It [faith] gives my life much more meaning and richness and a hope that I’d never had before” where “Faith to me is believing in something bigger than me” (FG3, V&PLD16, BN). Thus, intrinsic religiosity offered support for this participant.

## Theme 1: Subtheme 2 Help: God Can Act

God is also perceived as offering help as noted by one participant: “He actually really understands that I use eating disorder to cover pain and to help cope with…when life is hard” (FG2, PLD03, OE). God is perceived as someone “who wants the best for everybody and there is no judgement” (FG1, C11, AN) which mitigates the issue of guilt. One participant identified help from God through him “speaking to me”, “standing behind me…offering encouragement to get better” and “God’s sort of fighting my corner” (FG2, PLD08, BED). This highlighted how intrinsic religiosity enabled a deep connection that helped in relation to their eating disorder.

## Theme 1: Subtheme 3 Questions for God

Faith and relationships with God could be complicated and some individuals struggled to make sense of it. There were feelings of “guilt, that God is angry” (FG2, PLD03, OE) and prayer for healing that went unanswered: “I pray for it to go away, but it never does, and I don’t understand why” (FG2, PLD07, AN). Having an eating disorder also led to a sense of blocking their relationship with God “I’ve put God and Jesus at the forefront of everything in my life, and…something getting in the way of that in terms of my eating behaviours just kind of overwhelmed me” (FG4, PLD10, BED). In this case the eating disorder here acted as a barrier in their relationship with God (Homan & Boyatzis, 2010).

## Theme 2: Experiencing Relational Support from People

This theme focuses on the relationship and connection with other people, emphasising the community of those affected by eating disorders and the impact that others (such as participants in tastelife groups) have in relation to coping. One carer noted they “found the emotional support really helpful and we felt less alone” (FG1, C12, AN) This was reinforced by a participant who noted: “Other people have the same sorts of issues and problems as me and I’m not a freak” (FG2, PLD07, AN). The relational support was valuable whether the background was a person living with an eating disorder, carer or volunteer.

## Theme 2: Subtheme 1 Valuing Shared Experiences

Empathy and solidarity, where people shared their experiences and perspectives were often highly valued: “What I found so wonderful about tastelife was the fact that you were with people who knew how much pain this whole business causes” (FG1, PLD01, AN) and they continued people “knew what you were talking about and understood.” Irrespective of faith, these shared experiences were important, reinforcing a sense of togetherness and community.

## Theme 2: Subtheme 2 Better Together

Where people did not find themselves exactly in the “same boat”, different perspectives could be difficult to hear, even if they were helpful: “I would say mixing parents, carers and sufferers, I found quite hard…. because of that mixed content…it was helpful to see that other perspective…even though it felt uncomfortable” (FG2, PLD07, AN). This was the tastelife ethos of allowing everyone to participate in their programmes reflected in the focus group. Another participant suggested: “I didn’t shy away from it and I found it really useful to hear their perspectives…they’d get upset, they’d cry and it did make me think…see what are you doing to yourself” (FG2, PLD08, BED). Hearing other perspectives, whilst difficult, was helpful in relation to thinking about their eating disorder in the context of their immediate community.

## Theme 2: Subtheme 3 Making Space for the Presence of God: Exploring Spirituality and Faith in Recovery

The tastelife groups also gently offered the opportunity to explore the role of faith in relation to their eating disorder journeys. For example, one person noted their recovery concerned “recovery about my identity in Christ” (FG4, PLD10, BED) whilst another (FG1, PLD01, AN) “felt reconnected” with their faith in a manner similar to Richards et al. (2018) who noted a rediscovery of identity in Christ. The tastelife course enabled participants “to think about God, it started the process in my head” (FG4, PLD10, BED). One carer noted it was “very sensitively done and we were offered private prayer at the end if we wanted it, we could stay behind for that and for the sort of prayer and emotional support, but it wasn’t done in a forceful way at all…very gentle” (FG1, C11, AN). This was echoed by another carer who commented: “it wasn’t all about God and it certainly wasn’t thrust down our throats…it was very much very tentatively and gently there” (FG3, C13, OSFED). Prayer support also offers relational and emotional support from the group and deepening connections.

## Theme 3: Experiences of a Church Community: A Mixed Bag

Participants who belonged to a church community reported a variety of responses to their eating disorders. Some were positive and supportive, whilst others were less helpful and judgemental or simply ignoring existing issues.

## Theme 3: Subtheme 1 Care and Compassion: Prayerful Support

One participant noted they were deeply appreciative of compassionate support: “My church community I’ve got round me has been really compassionate and supportive” (FG2, PLD07, AN) and “praying [is] very useful because it almost takes the pressure off” (FG2, PLD07, AN), this included online support. Prayer support can be important “when I’ve been stronger in my faith and turned to prayer and help from others, instead of either undereating or overeating” (FG2, PLD05, BED). As Richards et al. (2018) also note, spirituality directly impacts treatment and recovery.

## Theme 3: Subtheme 2 Vulnerability: The Risk and Cost of Being Real

The cost of being real included feeling vulnerable: “You do feel a bit of an outcast” (FG2, PLD06, AN) and the feeling of not being understood by church communities where “Churches…not understanding mental health and eating disorders” (FG2, PLD05, BED). This impacts anxieties about their relationship with God where individuals can experience “an internal spiritual disconnection” (Smith et al., 2004: 178). Church and faith communities can exacerbate this feeling of disconnection.

## Theme 3: Subtheme 3 Best Intentions but Often Unhelpful Approaches

Best intentions were recognised but were not always helpful: “You know they can have the best intentions, but they just don’t know how to cope with you…it’s just to sweep it under the carpet” (FG2, PLD08, BED). Thus, church and church communities can sometimes exacerbate issues: “I’ve had big problems actually going to church and being in church…I have to keep a face on” (FG4, PLD09, AN). This can lead to a disconnection from God and faith (See Smith et al., 2004).

## Theme 4: Trusting and Widening the Community: A Ripple Effect

It was important to feel safe as part of a trusting community so that everyone felt able to speak: “I’m a really shy introverted person…I felt so safe in the group that I would literally just tell them how it was” (FG2, PLD08, BED). This spilled out into everyday life for many participants: “I’m speaking a little bit about it now with my mum and sister” (FG2, PLD08, BED) as a result. This highlights the importance of considering contexts in relation to recovery.

## Theme 4: Subtheme 1 Accountable and Encouaraging

The relationships formed during tastelife groups went far beyond official meetings. “We all keep in contact and meet up and having that kind of accountability where we’re continuing to encourage each other post session” (FG4, PLD10, BED) enabling the recovery journey to be built on shared understandings, shared experiences and shared ongoing conversations. Thus, the wider communities and relationships are important for these participants.

## Theme 4: Subtheme 2  Communication and Inclusion: Building Relationships

Similarly, one carer noted “there was a kind of GP who was part of running the course…. we had her number and could ring her from time to time” (FG1, C12, AN). Part of these relationships involved the notion that everyone was included “What really, really appealed about tastelife was the fact that my partner could come too and previously, you know, when I have been an outpatient in eating disorders, he’d been very much excluded from that” (FG1, PLD01, AN). The importance of including existing relationships, including those supporting someone with an eating disorder, can impact recovery.

## Theme 4: Subtheme 3 A Desire to Help Others

Many volunteers in the tastelife groups have themselves had an eating disorder. Their desire, motivated by empathy and compassion, is to offer hope to others to lead others through the path they have travelled and to help them get to the other side. “I travelled through part way recovery from bulimia and I’d always hoped and dreamed, particularly once I became a Christian years later, that maybe there’d be an opportunity and a door would open for me to perhaps help people in the community” (FG3, V&PLD16, BN). This relationship also gave a purpose that could lift them out of the illness to see a bigger picture in which personal experience can be used to help others. There is, however, a cost to helping others as noted by one volunteer in terms of “time commitment and the emotional commitment of walking along with people” (FG3, V17, NED).

## Theme 5: Experiencing Relationship Support from Social Media: “It Should Come with a Health Warning”

Social media is so embedded in our daily lives, connecting us with friends, family, celebrities and others. As one participant noted: “I love social media…its actually quite a big part of my life” (FG4, PLD10, BED). Another participant noted: “You can’t really avoid it…even on Facebook scrolling down, friends are constantly posting pictures of the food…people are posting things about you need to get back on my exercise regime and I’m feeling fat” (FG1, PLD02, BN). The time spent on social media may also impact existing issues as suggested by Padin et al. (2021).

## Theme 5 Subtheme 1: Helpful and Positive Social Media

Although social media is often perceived negatively, it can also play a positive role in relation to eating disorders enabling supportive communities online: “So I had a pop-up about something called second nature recently which is a sort of habit changing community…it’s been really helpful. Really positive community” (FG2, PLD07, AN). It can also help when people don’t know where to turn, “I feel we were really saved by the Feast website which is [an] International Eating Disorders website, and when I was at our lowest ebb, I just posted the desperate post “help” [and] became part of a closed Facebook group called “At the Dinner Table Forum” which was just incredible support from parents who had been through it and out the other end” (FG1, C12, AN). As Basterfield et al., (2018:605) note that “when technology is used to support recovery there can be positive outcomes for those with eating disorders” especially if it creates a community for meeting like-minded and supportive people.

## Theme 5 Subtheme 2: Unhelpful and Negative Social Media

At the same time, social media can be negative and exacerbate existing issues: “it just needs a warning label on it…this may damage your health…it just made me a lot worse and really increased my anxiety and you know, it [should]… come with a health warning” (FG1, PLD01, AN). This extends to groups that actively promote eating disorders as “it’s easy to find pro-anorexic groups and they try and pretend that they’re your friend but they’re not. They’re absolutely not” (FG1, PLD01, AN). Appearance focused media can be a cause for concern (Maes & Vandenbosch, 2022) and it can facilitate comparisons (Jarman et al., 2021). As one participant suggested “looking at other athletes pictures and their bodies and you know sort of comparing yourself…and that constant feeding of comparison” (FG2, PLD06, AN). As Meier and Gray (2017) suggest, social media provides more opportunities for such comparisons.

## Theme 5 Subtheme 3: Selective Use

Taking control of social media included taking time away from it: “I’ve’ had bad experiences in terms of social media and my eating disorder but now I stay off it” (FG2, PLD04, AN). This included limiting the amount of time spent on social media “I think that’s where for me I just try and keep things quite limited [to] what I’m choosing to see” (FG2, PLD06, AN). Some people chose to delete social media completely in order to take control (FG1, PLD02, BN) as noted by one participant: “I only have Facebook and I deleted it for about 5 years because I just found it overwhelming” but they went back on it to reconnect with people.

## Discussion

### Summary of Findings

The five main themes have addressed the four key research objectives, exploring religious issues and social media from those attending tastelife programmes. The findings from this study indicate the importance of relationships, in a variety of forms, to those attending the eating disorder recovery programme tastelife UK. The significance of relational support from God and the centrality of faith as a determining recovery factor is apparent. Religious coping was present in the subthemes in the form of help, hope and prayer support from a religious community, where there was a safe place to explore spirituality and faith. Religious and spiritual struggles were evidenced where eating disorders were considered as hindering an individual’s relationship with God and led to feelings of guilt. Crisis was apparent when individuals could not understand why God had not healed them when they prayed. Individuals also felt vulnerable in church settings that did not understand their condition. Our findings highlighted how social media could be positive, including access to religious communities whilst avoiding unhelpful social media use, but self-objectification in relation to social media use did impact participant’s eating disorders (Cottingham, 2014). Intrinsic religiousness also buffered against this impact and resulted in less body image dissatisfaction (Homan & Cavanaugh, 2013). Faith-based altruism, as well as past experiences with eating disorders, was a motivator for many volunteers to support tastelife UK participants and this could impact recovery.

### Consistency with Other Literature

Positive religious coping and God being a source of hope and help is consistent with wider evidence around the use of religious coping within health (Strenger et al., 2016). Epidemiological evidence shows religion as an important resource for many people struggling with illnesses (Harrison et al., 2001). Positive religious coping methods includes seeking help from God, seeing how God might be trying to use a situation, reflecting a secure relationship with a transcendent force, as well as a sense of spiritual connectedness with others, and a benevolent world view. Negative religious coping methods including doubting God’s love or power, wondering whether the situation is a punishment from God, reflect underlying spiritual tensions and struggles within oneself, with others, and with the divine (Pargament et al., 2000). The positive and negative aspects to religious coping may explain the association of religious coping with both positive and negative outcomes. There is evidence to suggest this may be the case with eating disorders (Rider et al., 2014) and struggles of those with disordered eating patterns or behaviour have been found in those with and without faiths (Exline et al., 2016).

Wider literature suggests that social identity formation in the eating disorder community is important but also problematic as it can threaten positive changes in personal identity (Kendal et al., 2017). This stems from the viewpoint that causes are located within the person (Malson et al., 2011) and making identity connections with others experiencing eating disorders tends to lead to unhelpful recovery behaviours (Vandereycken, 2011; Williams & Riley, 2013). Desired change in personal identity to promote recovery has been seen as happening at the intrapersonal level (Abbate-Daga et al., 2013), without taking social context and role of group membership into account. However, recovery can be enabled with the support and understanding of those in similar circumstances (Ison & Kent, 2010). Social identities can create resilience or vulnerability dependent upon contextual group norms and values (Cruwys & Gunasseelan, 2016: Dingle et al., 2015). The evidence in this study supports the view that people living with eating disorders, along with carers, found a social identity from connection through their eating disorder, which can lead to building resilience, support coping and recovery. Positive relationships that enable self-awareness and personal growth (Humbert, 2016) can help health and wellbeing in eating disorder recovery.

Consideration of the importance of understanding the person with an eating disorder within their social contexts of family, friends, social media and the wider community is important in creating a safe space for transformative dialogue (Richards et al., 2018) where the person is enabled to build a personal narrative of the problem in a supportive relationship characterised by respect and collaboration (Humbert, 2016). Online support groups can be positive (Leonard et al., 2015) with participants finding helpful communities to meet needs not being met in the offline environment (Branley & Covey, 2017). Despite positive benefits, social media still provides more opportunities for comparisons (Tiggemann & Anderberg, 2019) which can impact eating disorder pathology via body dissatisfaction (Santarossa & Woodruff, 2017; Wick & Keel, 2020). The theme of avoiding unhelpful use of social media was dominant amongst participants. Weinstein (2018) suggested that social media use is similar to a “seesaw” where different elements tip the balance positive or negatively. Helpful support groups, positive care, compassion and support from social media, as well as communities, church or faith contexts and God can move people living with eating disorders forward more positively on their eating disorder recovery journey. Negative groups and content, feelings of guilt, shame, church communities that do not understand the eating disorder, and a God perceived more negatively can lead to the recovery journey being impeded.

Care and compassion have intrinsic power to positively impact the eating disorder recovery journey (Stammers, 2020) but it is not often acknowledged in functional medical approaches. Conversely expectations of church members and perceiving eating disorder as *sin* can lead to negative impact on the eating disorder journey. If religious faith is important to the person living with an eating disorder, then the role it plays should be acknowledged (Cumella et al., 2007). As Basterfield et al., (2018:611) note that “future studies should investigate the use of technologies as an adjunct to intensive treatment.”

### Strengths and Limitations

This study draws on qualitative research from those who have participated in the eight-session community self-help group courses either as a person living with an eating disorder, carer or volunteer coordinator of a group in the UK. When interpreting results, it is important to address the nature of our sample. The participants were a relatively homogenous group, although diverse in terms of age and experiences, they identified as White, 16 identified as Christian and 1 as Buddhist all from the UK. It would be useful to explore focus groups with a mixture of ethnicities and also different faiths as King et al., (2019: 928) note that “body dissatisfaction and EDBs are of concern and are found in every ethnicity.” The focus groups (apart from focus group 2) were a mixture of carers, those living with an eating disorder and volunteers and whilst this was perceived to be beneficial it might be useful to explore focus groups consisting of solely carers and volunteers. The fact that there were only two males (both of whom were carers) may also mean that our findings are not transferable to males with eating disorders. As the focus groups were online this may have meant that discussions were not always as free flowing as they might be face to face and those who spoke up initially may have subsequently influenced others (see Vinokur & Burnstein, 1978), particularly if a specific type of eating disorder dominated.

The strengths of this study combine social media and religiosity with eating disorders and draws on the voices of those living with eating disorders including carers on the tastelife course. It highlights the importance of acknowledging faith(s) and social media in the role of recovery and the importance of relationships in each of these contexts. The qualitative nature of these focus groups enabled the voices of those living with eating disorders to illuminate their experiences in faith settings (churches), their local communities and social media, as well as exploring how a perceived relationship with God impacted on recovery. The sharing of complex circumstances (carer, person living with an eating disorder, volunteer) actually encouraged discussion in the focus groups, and enabled deeper sharing and wider perspectives to be explored. Whilst acknowledging that these are complex cultural contexts, the interaction of these settings was acknowledged in this research.

### Implications of Findings for Addressing Religious and Spiritual Issues in Eating Disorders

Implication of findings have been framed in the context of AACTT (Action, Actor, Context, Target, Time) (Presseau et al., 2019) to support readers to take action.

## Authors’ Recommendations for Individuals and Carers

Action: For Carers to be involved in supporting recovery and individuals to have the opportunity to explore spirituality, faith and social media in their recovery. Resources to be made available on social media.

Actor: Individuals and carers.

Context: Social media and support groups.

Target: People living with eating disorders and those caring and supporting those living with eating disorders.

Time: When the carer/individual requests this and wants to engage with social media.

## Small Groups, Faith-Based Communities and Healthcare Providers

Action**:** Need to be more prepared to respond appropriately to those with eating disorders to address perceived vulnerabilities including spiritual support groups in different environments and contexts.

Actor: Faith based communities, small groups, healthcare providers.

Context: Community context, small groups, Churches and religious institutions, clinical contexts.

Target: Education in church contexts to understand the addictive nature of eating disorders and how recovery can be effected by social media and intrinsic/extrinsic religious motivations.

Time: Intervention by skilled people of faith during recovery programmes and on social media.

## References

[CR1] Abbate-Daga G, Amianto F, Delsedime N, De-Bacco C, Fassino S (2013). Resistance to treatment and change in anorexia nervosa [corrected]: A clinical overview. BMC Psychiatry..

[CR2] Akrawi D, Bartrop R, Potter U, Touyz S (2015). Religiosity, spirituality in relation to disordered eating and body image concerns: A systematic review. Journal of Eating Disorders.

[CR3] Basterfield A, Dimitropoulos G, Bills D, Cullen O, Freeman V (2018). “I would love to have online support but I don’t trust it”: Positive and negative views of technology from the perspective of those with eating disorders in Canada. Health and Social Care in the Community..

[CR4] Beat Eating Disorders. (2022). Statistics for Journalists*. BEAT*. https://www.beateatingdisorders.org.uk/media-centre/eating-disorder-statistics/

[CR5] Beattie P, Bettache K, Chong KCY (2019). Who is the Neoliberal? Exploring Neoliberal beliefs across East and West. Journal of Social Issues.

[CR6] Blodgett Salafia EH, Jones ME, Haugen EC, Schafer MK (2015). Perceptions of the causes of eating disorders: A comparison of individuals with and without eating disorders. Journal of Eating Disorders..

[CR7] Boyatzis C, Kline S, Backof S (2007). Experimental evidence that theistic-religious body affirmations improve women’s body image. Journal for the Scientific Study of Religion..

[CR8] Branley DB, Covey J (2017). Pro-ana versus Pro-recovery: A content analytic comparison of social media users’ communication about eating disorders on twitter and tumblr. Frontiers in Psychology.

[CR9] Braun V, Clarke V (2006). Using thematic analysis in psychology. Qualitative Research in Psychology.

[CR10] Brotsky SR, Giles D (2007). Inside the “Pro-ana” community: A covert online participant observation. Eating Disorders the Journal of Treatment & Prevention..

[CR11] Bryman A (2016). Social research methods.

[CR12] Burnette CB, Kwitowski MA, Mazzeo SE (2017). I don’t need people to tell me I’m pretty on social media. A quantitative study of social media and body image in early adolescent girls. Body Image.

[CR13] Byrne D (2022). A worked example of Braun and Clarke’s approach to reflexive thematic analysis. Quality & Quantity..

[CR14] Carrotte ER, Vella AM, Lim MS (2015). Predictors of “Liking” three types of health and fitness-related content on social media: A cross-sectional study. Journal of Medical Internet Research..

[CR15] Chatard A, Bocage-Barthélémy Y, Selimbegović L, Guimond S (2017). The woman who wasn’t there: Converging evidence that subliminal social comparison affects self-evaluation. Journal of Experimental Social Psychology..

[CR16] Cottingham ME, Davis L, Crycraft A, Keiper CD, Abernethy AD (2014). Disordered eating and self-objectification in college women: clarifying the roles of spirituality and purpose in life. Mental Health, Religion & Culture..

[CR17] Cruwys T, Gunaseelan S (2016). “Depression is who I am”: Mental illness identity, stigma and wellbeing. Journal of Affective Disorders..

[CR18] Cumella EJ, Kally Z, Wall AD (2007). Treatment responses of inpatient eating disorder women with and without co-occurring obsessive-compulsive disorder. Eating Disorders..

[CR19] Dingle GA, Cruwys T, Frings D (2015). Social identities as pathways out of addiction. Frontiers in Psychology..

[CR20] Exline JJ, Homolka SJ, Harriott VA (2016). Divine struggles: Links with body image concerns, binging, and compensatory behaviours around eating. Mental Health, Religion & Culture.

[CR21] Fardouly J, Vartanian LR (2016). Social media and body image concerns: Current research and future directions. Current Opinion in Psychology..

[CR22] Forthun LF, Pidcock BW, Fischer JL (2003). Religiousness and disordered eating: Does religiousness modify family risk?. Eating Behaviours..

[CR23] Fusch PI, Ness LR (2015). Are we there yet? Data saturation in qualitative research. The Qualitative Report.

[CR24] Geller S, Handelzalts J, Gelfat R, Arbel S, Sidi Y, Levy S (2018). Exploring body image, strength of faith, and media exposure among three denominations of Jewish Women. Current Psychology.

[CR25] Goulet C, Henrie J, Szymanski L (2017). An exploration of the associations among multiple aspects of religiousness, body image, eating pathology, and appearance investment. Journal of Religion and Health.

[CR26] Griffiths CA, Ryan P, Foster JH (2011). Thematic analysis of Antonovsky’s sense of coherence theory. Scandinavian Journal of Psychology.

[CR27] Hammarberg K, Kirkman M, DeLacey S (2016). Qualitative research methods: When to use them and how to judge them. Human Reproduction.

[CR28] Harrison MO, Koenig HG, Hays JC, Eme-Akwari AG, Paragement KI (2001). The epidemiology of religious coping: A review of recent literature. International Review of Psychiatry.

[CR29] Hauck C, Cook B, Ellrott T (2020). Food addiction, eating addiction and eating disorders. Proceedings of the Nutrition Society..

[CR30] Homan KJ, Boyatzis.  (2010). The protective role of attachment to god against eating disorder risk factors: Concurrent and prospective evidence. Eating Disorders..

[CR31] Homan KJ, Cavanaugh BN (2013). Perceived relationship with God fosters positive body image in College Women. Journal of Health Psychology..

[CR32] Humbert TK (2016). Spirituality and occupational therapy.

[CR33] National Institute for Health and Care Excellence (NICE). (2017a). Clinical guideline [CG89] (2017a). *Child maltreatment: when to suspect maltreatment in under 18s,* National Institute for Health and Care Excellence. 1–35. https://www.nice.org.uk/guidance/cg89/resources/child-maltreatment-when-to-suspect-maltreatment-in-under-18s-pdf-975697287109. 31999415

[CR34] Ison J, Kent S (2010). Social Identity in eating disorders. European Eating Disorders Review..

[CR35] Jarman HK, McLean SA, Slater A, Marques MD, Paxton SJ (2021). Direct and indirect relationships between social media use and body satisfaction: A prospective study among adolescent boys and girls. New Media & Society.

[CR36] Karsay K, Trekels J, Eggermont S, Vandenbosch L (2021). “I (Don’t) Respect My Body”: Investigating the role of mass media use and self-objectification on adolescents’ positive body image in a cross-national study. Mass Communication and Society..

[CR37] Kendal S, Kirk S, Elvey R, Catchpole R, Pryjmachuk S (2017). How a moderated online discussion forum facilitates support for young people with eating disorders. Health Expectations.

[CR38] King LH, Abernethy AD, Keiper C, Craycraft A (2019). Spirituality and eating disorder risk factors in African American women. Eating and Weight Disorders Studies on Anorexia, Bulimia and Obesity..

[CR39] Knobloch-Westerwick S, Kennard AR, Westerwick A, Willis LE, Gong V (2014). A crack in the crystal ball? Prolonged exposure to media portrayals of social roles affect possible future selves. Communication Research..

[CR40] Lee YJ (2018). Is your church “liked” on Facebook? Social media use of Christian congregations in the united States. Nonprofit Management & Leadership.

[CR41] Leonard K, Quesenberry AC, Lindsay JM (2015). Moderated social media support groups for patients. Journal of Consumer Health on the Internet..

[CR42] Mabe A, Forney KJ, Keel PK (2014). Do you “like” my photo? Facebook use maintains eating disorder risk. International Journal of Eating Disorders..

[CR43] Maes C, Vandenbosch L (2022). Adolescent girls’ Instagram and TikTok use: Examining relations with body image-related constructs over time using random intercept cross-lagged panel models. Body Image.

[CR44] Maguire M, Delahunt B (2017). Doing a thematic analysis: A practical, step-by-step guide for learning and teaching scholars. All Ireland Journal of Higher Education.

[CR45] Malson H, Bailey L, Clarke S, Treasure J, Anderson G, Kohn M (2011). Un/imaginable future selves: a discourse analysis of in-patients’ talk about recovery from an ‘eating disorder’. European Eating Disorder Review.

[CR46] Marchetti-Mercer M (2012). Those easily forgotten: The impact of emigration on those left behind. Family Process.

[CR47] Marsden P, Karagianni E, Morgan JF (2007). Spirituality and clinical care in eating disorders: A qualitative study. International Journal of Eating Disorders.

[CR48] McClean SA, Paxton SJ, Wertheim EH, Masters J (2015). Selfies and social media: Relationships between self-image editing and photo-investment and body dissatisfaction and dietary restraint. Journal of Eating Disorders.

[CR49] Meier EP, Gray J (2017). Facebook photo activity associated with body image disturbance in adolescent girls. Cyberpsychology, Behavior and Social Networking..

[CR50] Mingola J, Hutchinson AD, Wilson C, Gleaves DH (2017). The relationship between social networking site use and the internalisation of a thin ideal in females: A meta-analytic review. Frontiers in Psychology..

[CR51] Mitra, B. (2017) Written evidence submitted to the youth select committee (body image and eating disorders) 012, *British Youth Council -Youth Select Committee 2018: A Body Confident future.* 1–47. https://byc2016.wpenginepowered.com/wp-content/uploads/2017/11/Youth-Select-Committee-A-Body-Confident-Future.pdf

[CR52] Mitra B, Taylor L, Milburn-Curtis C, McCarron J (2018). Gendering Worcester news. Journal of the Association for Journalism Education..

[CR53] Mitra B, Webb M, Wolfe C (2014). Audience responses to the physical appearance of television newsreaders. Participations, Journal of Audience and Reception Studies..

[CR54] National Institute for Health and Care Excellence (NICE). (2017b). guideline [NG69] (2017). *Eating disorders: recognition and treatment*, National Institute for Health and Care Excellence. 1–45. https://www.nice.org.uk/guidance/ng69/resources/eating-disorders-recognition-and-treatment-pdf-1837582159813.

[CR55] National Health Service (NHS). (2021). Overview – Eating disorders. *National Health Service.* https://www.nhs.uk/mental-health/feelings-symptoms-behaviours/behaviours/eating-disorders/overview/.

[CR56] National Eating Disorders Association (NEDA). (2022). National Eating Disorders Association. *General Statistics. *https://www.nationaleatingdisorders.org/get-involved/nedawareness.

[CR57] Oman D (2018). Why religion and spirituality matter for public health.

[CR58] Padin PF, González-Rodriguez R, Verde-Diege C, Vázquez-Pérez R (2021). Social media and eating disorders psychopathology: A systematic review. Cyberpsychology: Journal of Psychosocial Research on Cyberspace.

[CR59] Palmberg AA, Stern M, Kelly NR, Bulik C, Belgrave FZ, Trapp SK, Hofmeier SM, Mazzeo SE (2014). Adolescent girls and their mothers talk about experiences of binge and loss of control eating. Journal of Child and Family Studies.

[CR60] Pargament KI, Koenig HG, Perez LM (2000). The many methods of religious coping: Development and initial validation of the RCOPE. Journal of Clinical Psychology.

[CR61] Perloff RM (2014). Social media effects on young women’s body image concerns: Theoretical perspectives and an agenda for research. Sex Roles.

[CR62] Presseau J, McCleary N, Lorencatto F, Patey AM, Grimshaw JM, Francis JJ (2019). Action, actor, context, target, time (AACTT): A framework for specifying behaviour. Implementation Science.

[CR63] Priory (2022). Eating Disorder Statistics. *Priory group.* https://www.priorygroup.com/eating-disorders/eating-disorder-statistics

[CR64] Reczek C (2014). Conducting a multi family member interview. Family Process.

[CR65] Richards, P. S., Weinberger-Litman, S. L., Susov, S., & Berrett, M. E. (2013). Religiousness and spirituality in the etiology and treatment of eating disorders. In K. I. Pargament, A. Mahoney, & E. P. Shafranske (Eds.), *APA handbook of psychology, religion, and spiritua*lity (Vol. 2): An applied psychology of religion and spirituality, 319–333. American Psychological Association. 10.1037/14046-016

[CR66] Richards PS, Berrett ME, Hardman RK, Eggett DL (2007). Comparative efficacy of spirituality, cognitive, and emotional support groups for treating eating disorder inpatients. Eating Disorders: The Journal of Treatment & Prevention.

[CR67] Richards PS, Caoili CC, Crowton SA, Berrett ME, Hardman RK, R. K., Jackson, R. N., & Sanders, P. W.  (2018). An exploration of the role of religion and spirituality in the treatment and recovery of patients with eating disorders. Spirituality in Clinical Practice.

[CR68] Rider KA, Terrell DJ, Sisemore TA, Hecht JE (2014). Religious coping style as a predictor of the severity of anorectic symptomology. Eating Disorders.

[CR69] Rosenvinge JH, Pettersen G (2015). Epidemiology of eating disorders, part I: Introduction to the series and a historical panorama. Advances in Eating Disorders.

[CR70] Saffran K, Fitzsimmons-Craft EE, Kass AE, Wilfley DE, Barr Taylor C, Trockel M (2016). Facebook usage amongst those who have received treatment for an eating disorder in a group setting. International Journal of Eating Disorders..

[CR71] Saiphoo AN, Halevi LD, Vahedi Z (2020). Social networking site use and self-esteem: A meta-analytic review. Personality and Individual Differences..

[CR72] Santarossa, S. & Woodruff, S.J. (2017). #Social media: Exploring the relationship of social networking sites on body image, self-esteem, and eating disorders. *Social Media + Society,* 3(2), 1–10. 10.1177/2056305117704407

[CR73] Slater A, Tiggemann M (2015). Media exposure, extracurricular activities, and appearance-related comments as predictors of female adolescents’ self-objectification. Psychology of Women Quarterly..

[CR74] Smith MH, Richards PS, Maglio CJ (2004). Examining the relationship between religious orientation and eating disturbances. Eating Behaviours.

[CR75] Spangler DL (2010). Heavenly bodies: Religious issues in cognitive behavioral treatment of eating disorders. Cognitive and Behavioral Practice.

[CR76] Spettigue W, Henderson KA (2004). Eating disorders and the role of the media. The Canadian Child and Adolescent Psychiatry Review..

[CR77] Stammers HR (2020). The theological language of anorexia: An argument for greater rapproachement between chaplains and physicians. Feminist Theology.

[CR78] Stice E, Gau JM, Rhode P, Shaw J (2018). Risk factors that predict future onset of each DSM-5 eating disorder: Predictive specificity in high-risk adolescent females. Journal of Abnormal Psychology.

[CR79] Strenger AM, Schnitker SA, Felke TJ (2016). Attachment to God moderates the relation between sociocultural pressure and eating disorder symptoms as mediated by emotional eating. Mental Health, Religion & Culture.

[CR80] Teufel M, Hofer E, Junne F, Sauer H, Zipfel S, Giel KE (2013). A comparative analysis of anorexia nervosa groups on Facebook. Eating and Weight Disorders.

[CR81] Thornton LM, Mazzeo SE, Bulik CM (2011). The heritability of eating disorders: Methods and current findings. Current Topics in Behavioral Neurosciences.

[CR82] Tiggemann M, Anderberg I (2019). Social media is not real; The effect of ‘Instagram vs reality’ images on women’s social comparison and body image. New Media and Society.

[CR83] Tong A, Sainsbury P, Craig J (2007). Consolidated criteria for reporting qualitative research (COREQ): A 32-item checklist for interviews and focus groups. International Journal for Quality in Health Care.

[CR84] Vandereycken W (2011). Media hype, diagnostic fad or genuine disorder? Professionals’ opinions about night eating syndrome, orthorexia, muscle dysmorphia and emetophobia. Eating Disorders..

[CR85] Victor CGP, Treschuk JV (2020). Critical literature review on the definition clarity of the concept of faith, religion, and spirituality. Journal of Holistic Nursing.

[CR86] Vinokur A, Burnstein E (1978). Depolarization of attitudes in groups. Journal of Personality and Social Psychology..

[CR87] Weinstein E (2018). The social media see-saw: Positive and negative influence on adolescents’ affective well-being. New Media & Society.

[CR88] Wick MR, Keel PK (2020). Posting editing photos of the self: Increasing eating disorder risk or harmless behaviour?. Journal of Eating Disorders..

[CR89] Wilksch S, O’Shea A, Ho P, Byrne S, Wade TD (2020). The relationship between social media use and disordered eating in young adolescents. International Journal of Eating Disorders..

[CR90] Williams, C. & Riley, S. (2013). Finding Support and Negotiating Identity: An Analysis of the Structure and Content of Newbie Posts and their Elicited Replies on Five Pro-Eating Disorder Websites. *Social Science Research on the Internet,* 1(2). 10.4000/reset.117

[CR91] Williams RJ, Ricciardelli LA (2014). Social Media and Body Image concerns: Further considerations and broader perspectives. Sex Roles.

